# Ecological response of an umbrella species to changing climate and land use: Habitat conservation for Asiatic black bear in the Sichuan‐Chongqing Region, Southwestern China

**DOI:** 10.1002/ece3.10222

**Published:** 2023-06-26

**Authors:** Yunchuan Dai, Heqing Huang, Yu Qing, Jiatong Li, Dayong Li

**Affiliations:** ^1^ Institute for Ecology and Environmental Resources, Research Center for Ecological Security and Green Development Chongqing Academy of Social Sciences Chongqing China; ^2^ Chongqing Academy of Ecology and Environmental Sciences Chongqing China; ^3^ Chongqing Industry Polytechnic College Chongqing China; ^4^ School of Tourism Kaili University Kaili China; ^5^ Key Laboratory of Southwest China Wildlife Resources Conservation (Ministry of Education) China West Normal University Nanchong China

**Keywords:** climate adaptation, climate refugia, dispersal path, *Ursus thibetanus*, vulnerability assessment

## Abstract

Climate and land use changes are increasingly recognized as major threats to global biodiversity, with significant impacts on wildlife populations and ecosystems worldwide. The study of how climate and land use changes impact wildlife is of paramount importance for advancing our understanding of ecological processes in the face of global environmental change, informing conservation planning and management, and identifying the mechanisms and thresholds that underlie species' responses to shifting climatic conditions. The Asiatic black bear (*Ursus thibetanus*) is a prominent umbrella species in a biodiversity hotspot in Southwestern China, and its conservation is vital for safeguarding sympatric species. However, the extent to which this species' habitat may respond to global climate and land use changes is poorly understood, underscoring the need for further investigation. Our goal was to anticipate the potential impacts of upcoming climate and land use changes on the distribution and dispersal patterns of the Asiatic black bear in the Sichuan‐Chongqing Region. We used MaxEnt modeling to evaluate habitat vulnerability using three General Circulation Models (GCMs) and three scenarios of climate and land use changes. Subsequently, we used Circuit Theory to identify prospective dispersal paths. Our results revealed that the current area of suitable habitat for the Asiatic black bear was 225,609.59 km^2^ (comprising 39.69% of the total study area), but was expected to decrease by −53.1%, −49.48%, and −28.55% under RCP2.6, RCP4.5, and RCP8.5 projection scenarios, respectively. Across all three GCMs, the distribution areas and dispersal paths of the Asiatic black bear were projected to shift to higher altitudes and constrict by the 2070s. Furthermore, the results indicated that the density of dispersal paths would decrease, while the resistance to dispersal would increase across the study area. In order to protect the Asiatic black bear, it is essential to prioritize the protection of climate refugia and dispersal paths. Our findings provide a sound scientific foundation for the allocation of such protected areas in the Sichuan‐Chongqing Region that are both effective and adaptive in the face of ongoing global climate and land use changes.

## Graphical abstract

Black bears' distribution range, dispersal paths, climate refugia, and priority protected area were identified; black bears would be negatively affected by the climate and land use change; black bears' distribution range would shrink and shift to high altitudes; climate and land use change would degrade black bears' dispersal paths.

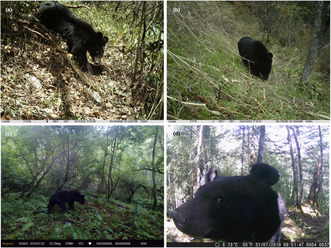

## 1 INTRODUCTION

Climate change has emerged as a significant threat to global biodiversity (Choi et al., 2021; Sun et al., 2023; Thomas et al., 2004; Urban, 2015). Climate change can impact species populations, through mechanisms such as range shifts, changes in abundance, and changes in phenology (Ali et al., 2021; Chen et al., 2011; Parmesan, 2006; Sauve et al., 2019; Stuart‐Smith et al., 2013; Thackeray et al., 2016). For instance, it can directly or indirectly affect the reproductive success rate of mammals by altering their food resources, habitats, and dispersal conditions (Bronson, 2009; Yom‐Tov, 2001). In addition, climate change can create new opportunities for invasive species to establish themselves and thrive in new habitats, which can have negative impacts on native ecosystems and biodiversity (Cheng et al., 2017). Under the influence of climate change, species' geographic distribution range will also alter (Dai et al., 2021). Species will migrate to areas with suitable habitats as climate conditions shift (Chen et al., 2011). Currently, a substantial amount of evidence shows that climate change has impacted the abundance and distribution range of species in various groups (Chen et al., 2011; Pimm et al., 2014). Climate change can also indirectly affect the distribution of species through biological factors related to the species (Tape et al., 2016). For example, vegetation not only provides suitable habitats for wildlife but also offers food resources. When the distribution of vegetation changes due to climate, the geographic distribution of related animals will also change (Boelman et al., 2015). Similarly, when the food chain relationship is affected by climate change, animals will adjust their distribution range (Harsch et al., 2009; Parmesan & Yohe, 2003).

In recent years, the impacts of climate change on wildlife populations, particularly carnivore species, have garnered increasing attention (Johnson et al., 2023; Rather et al., 2020; Usman et al., 2023). Recognizing the effects of these environmental shifts on carnivores is essential for comprehending the ecological implications of climate change (Dai et al., 2021). Notably, climate change can induce significant alterations in habitat suitability, disrupt the distribution and abundance of prey, and modify interactions among sympatric carnivores (Parmesan & Yohe, 2003; Post et al., 2019). These climate‐driven changes in habitat can fragment once suitable territories, impose limitations on movement corridors, and disturb established home ranges for carnivores (Mateo‐Sánchez et al., 2015; McGuire et al., 2016). As a result, the spatial distribution of carnivores can undergo shifts, leading to heightened overlap and the potential for resource competition among coexisting species. Furthermore, the availability and distribution of prey can be directly influenced by climate change, subsequently impacting the foraging behavior and energetic demands of carnivores, which further intensifies resource competition (Khosravi et al., 2021; Learmonth et al., 2006). Thus, comprehending the implications of climate change on carnivore communities is vital for unraveling the intricate ecological dynamics and potential cascading effects within ecosystems.

However, the impact of climate change on species' distribution ranges can vary greatly due to differences in the dispersal ability, migration speed, and direction of individual species. As such, changes in distribution range are often species‐specific (Parmesan & Yohe, 2003). Additionally, the impact of climate change on the suitable habitat ranges of different species is also species‐specific, resulting in some species experiencing range contraction while others experience range expansion (Pacifici et al., 2015; Sunday et al., 2011). These variations in the impact of climate change on different species' suitable habitat ranges can be attributed to their varying sensitivities based on their functional characteristics, including physiological traits, life‐history strategies, and biotic interactions (Lavergne et al., 2010). The connectivity of climatically suitable habitats plays a vital role in determining whether a species can successfully migrate to future suitable habitat patches and keep up with the pace of climate change (Hannah et al., 2007; Lawler et al., 2009). The capacity of a species to move to future climatically suitable habitats is primarily contingent on the connectivity of the habitat landscape and the dispersal ability of its population (Urban et al., 2012). However, improving the dispersal ability of populations is an evolutionary process that cannot be achieved in a short period of time (Early & Sax, 2011). Therefore, improving the landscape connectivity between suitable habitats of species is the optimal solution for protecting habitat integrity under the background of climate change.

Understanding how species respond to climate change is critical for increasing the effectiveness of conservation efforts and identifying habitats that are vulnerable to climate change for biodiversity conservation (Duffy et al., 2017; Parmesan, 2006). Species Distribution Models (SDMs) are valuable tools used to simulate suitable habitats for species. They can assess the vulnerability of habitats to climate change, predict how species will respond to large‐scale environmental changes, and help minimize the risk of species extinction (Elith & Leathwick, 2009; Guisan et al., 2017). In recent years, SDMs have successfully addressed various issues in ecology, conservation biology, biogeography, climate change, and evolutionary biology (Franklin, 2010; Phillips & Dudík, 2008). SDMs have become critical research tools across multiple disciplines and fields. These models have been used in numerous studies to assess suitable habitats for various endangered wildlife species, including vulnerability analyses, protection gap analyses, and identification of climate refugia (Bosso et al., 2022; Dai et al., 2019; Li et al., 2019, 2021). Significant progress has been made in wildlife management and conservation practices by simulating potential dispersal paths for species using Circuit Theory modeling and designing ecological networks (An et al., 2021; Dickson et al., 2019).

**FIGURE 1:**
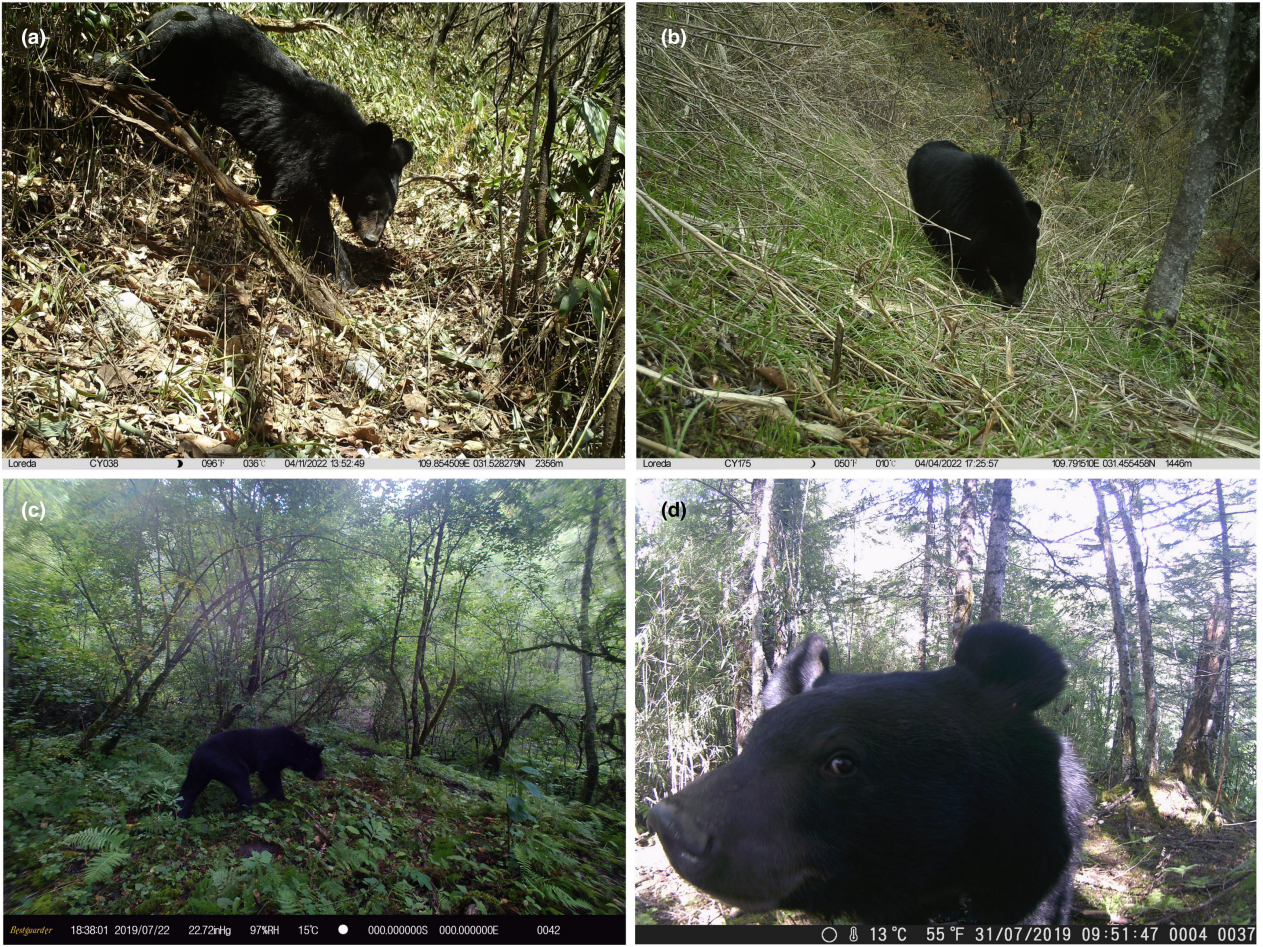
The Asiatic black bears (*Ursus thibetanus*) captured by camera traps. (a, b) were captured in the Yintiaoling National Nature Reserve, Chongqing municipality, China; (c) was captured in the Baihe National Nature Reserve, Sichuan province, China; (d) was captured in the Wanglang National Nature Reserve, Sichuan province, China.

The Asiatic black bear (*Ursus thibetanus*; Figure 1) belongs to the family Ursidae of the order Carnivora. It is listed as a vulnerable species on the IUCN Red List, is included in Appendix S1 of the Convention on International Trade in Endangered Species of Wild Fauna and Flora (CITES), and is a Class II‐protected species in China. The estimated population of wild Asiatic black bears in China is relatively low, with only 12,000–18,000 individuals and a maximum of 20,000 (Ma et al., 1994). Asiatic black bears are known to widely distribute throughout Southwest China, where the region plays an essential role in supporting their habitat and survival (Liu et al., 2009). The wild Asiatic black bears face numerous survival threats, which have had devastating effects on their populations. One significant threat to black bears is the trade of bear bile, which has been traditionally valued in some cultures for its medicinal properties (Mills & Servheen, 1994). The extraction of bear bile typically involves cruel and inhumane methods, such as keeping bears in captivity in so‐called bear farms. This practice leads to the poaching and killing of thousands of bears annually, severely impacting wild bear populations (Mills & Servheen, 1994; Sathyakumar, 2001; Ullah et al., 2020). The excessive exploitation of bears for their bile not only poses immediate risks to individual bears but also disrupts the natural balance of bear populations within their ecosystems. Furthermore, the trade in bear bile also threatens the habitats of black bears. Poachers and illegal traders often operate in protected areas, encroaching upon critical bear habitats and disturbing the delicate ecological balance (Sathyakumar, 2001). The loss of habitat due to human encroachment is another significant factor contributing to the decline in black bear populations (Ahmad et al., 2022; Beckmann & Berger, 2003; Goursi et al., 2021). As human populations expand, urbanization, deforestation, and industrial development encroach upon the once undisturbed territories of black bears, leading to habitat fragmentation and degradation (Ahmadzadeh et al., 2008). To protect wildlife, the Chinese government has implemented a range of laws and policies, notably the Wildlife Protection Law, while also actively engaging in international agreements such as CITES to address the global conservation of endangered species.

Although wildlife conservation laws have contributed to some degree of restoration in the population of the Asiatic black bear, there is still a lack of comprehensive ecological research on this species. Scientists have conducted extensive research on habitat selection (Liu et al., 2009; Takahata et al., 2013), stress reaction (Malcolm et al., 2014), human–bear conflict (Liu et al., 2011), and activity patterns (Zahoor, Liu, Wu, et al., 2021). While notable progress has been made in the field of Asiatic black bear conservation, there is a paucity of quantitative studies that have effectively investigated the complex relationship between the bear's habitat, climate change, and human activities. Without such analyses, it is difficult to accurately assess the conservation status of the bear's habitat and effectively plan for its long‐term protection (Foden et al., 2019; Preston et al., 2011). In our study, we established a suitable habitat distribution model based on distribution information, bioclimatic factors, geographic environmental factors, and human disturbance factors, and used Circuit Theory modeling to simulate the connectivity and potential dispersal paths between suitable Asiatic black bear habitats under current and future climate scenarios. The research objectives of this study include:

- Modeling the current suitable habitat and predicting suitable habitat in the 2070s. We aim to model the current suitable habitat for Asiatic black bears in the Sichuan‐Chongqing Region and make predictions for the suitable habitat in the 2070s. By utilizing climate data and ecological modeling techniques, we aim to understand how the bear's habitat may change over time due to climate change. This analysis will provide insights into the potential impacts on the species' distribution and help identify areas that may become unsuitable or suitable in the future.
- Assessing habitat vulnerability and identifying climate refugia. In addition to predicting future habitat suitability, our study seeks to assess the vulnerability of the bear's habitat to climate change. By evaluating various environmental factors and their potential impacts on the bear's habitat, we aim to identify areas that may serve as climate refugia. These refugia could potentially provide suitable conditions for the species to persist and adapt in the face of changing climatic conditions.
- Identifying potential corridors for Asiatic black bear migration. Understanding the connectivity between current and future suitable habitat is crucial for the long‐term survival of the Asiatic black bear population in the region. Our study aims to identify potential migration corridors that could facilitate the movement of bears from their current habitat to suitable habitat in the 2070s. Identifying these corridors will aid in the development of conservation plans, allowing us to prioritize habitat protection and management strategies along the migration routes.

The ultimate goal of our study is to provide a scientific basis for formulating effective conservation plans for the Asiatic black bear in the Sichuan‐Chongqing Region, considering the backdrop of climate change. By combining our findings on habitat modeling, vulnerability assessment, and corridor identification, we hope to contribute valuable information to conservation practitioners and policymakers. Our research intends to support proactive conservation efforts aimed at mitigating the potential negative impacts of climate change on this endangered species.

## 2 MATERIALS AND METHODS

### 2.1 Study area and species occurrence data

**FIGURE 2:**
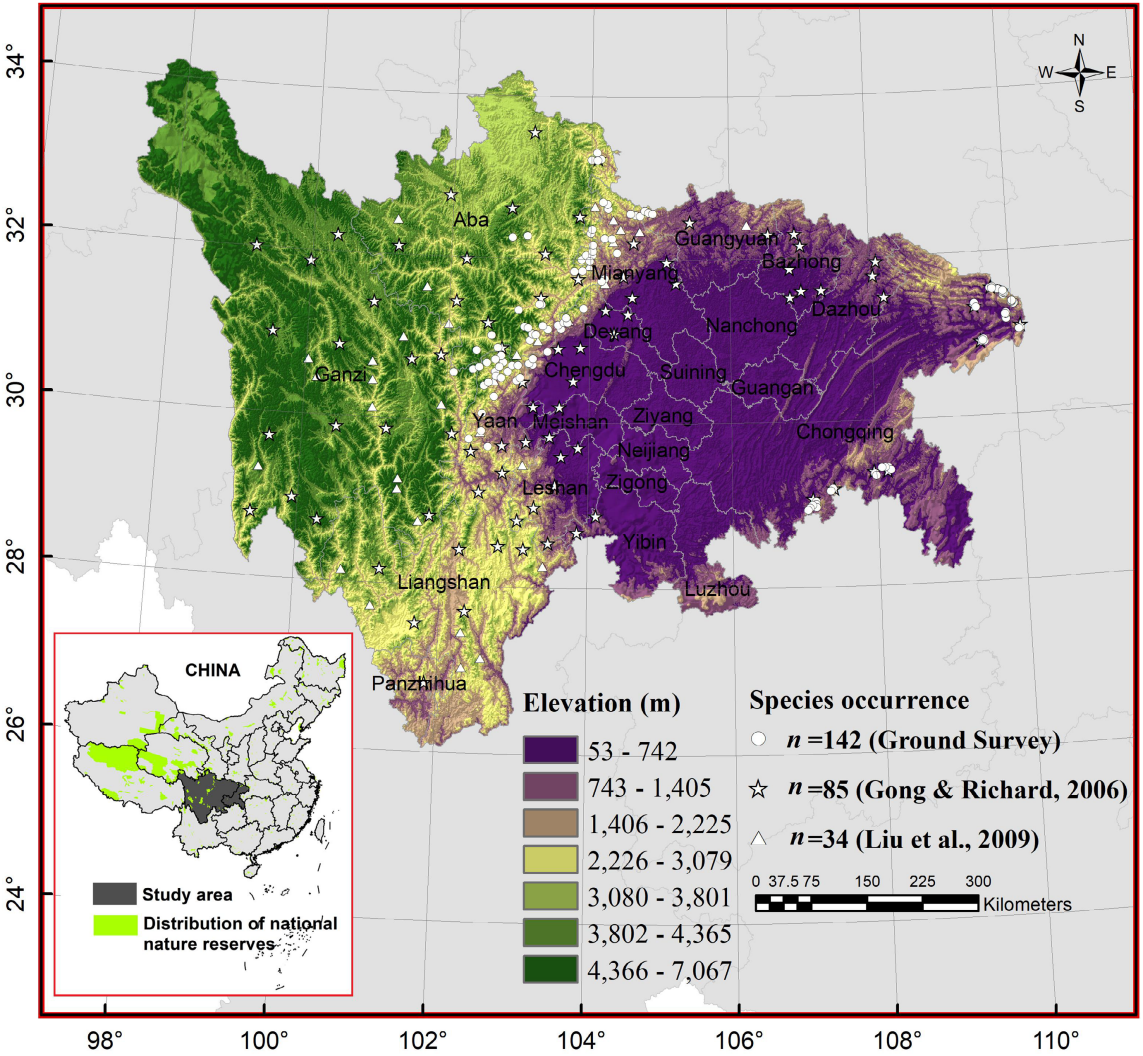
Location of study area and occurrence points of Asiatic black bear (Ursus thibetanus).

The Sichuan‐Chongqing Region (approximately 568,400 km^2^), comprising Sichuan Province and Chongqing Municipality, is a highly biodiverse area of Southwestern China (Figure 2). It boasts a diverse topography and varied climate types, encompassing several habitat types such as high mountains, hills, and plains. Due to its unique geographic environment and climate conditions, the Sichuan‐Chongqing Region hosts a wide range of vegetation types, including coniferous forests, broad‐leaved forests, alpine meadows, and grasslands. These habitats provide extensive living spaces and food sources for wildlife, creating favorable conditions for the survival and reproduction of numerous animal and plant species, making the area one of the global hotspots for biodiversity (Zhang et al., 2011). The region includes many rare and endangered species, such as the giant panda (*Ailuropoda melanoleuca*), golden monkey (*Rhinopithecus roxellana*), Asiatic black bear, brown bear (*Ursus arctos pruinosus*), and leopard (*Panthera pardus*).

One of the primary reasons for our focus on the Sichuan‐Chongqing Region is the availability of reliable data on black bear distribution in these specific areas. Our research team has dedicated extensive efforts to studying black bears in the Sichuan‐Chongqing Region, resulting in a rich dataset that encompasses various ecological factors and habitat characteristics unique to this region. While we recognize that suitable habitat areas extend beyond the political boundaries of the Sichuan‐Chongqing Region, our study aims to provide comprehensive insights into the specific context of black bears within this region. We understand that the distribution of suitable habitats transcend administrative divisions. However, by focusing on the Sichuan‐Chongqing Region, we can delve deeper into understanding the intricacies and nuances of black bear ecology, behavior, and habitat requirements in this particular geographic area. Our dataset includes a total of 261 GPS coordinates of Asiatic black bears. Of these, 142 coordinates were collected through ground surveys conducted between 2019 and 2022 (recorded from infrared camera traps, as well as identifying coordinates of presence through feces, footprints, hair, and foraging traces). An additional 119 coordinates were obtained from published literature and reports (Gong & Richard, 2006; Liu et al., 2009; Figure 2), contributing to the overall robustness of our dataset. To mitigate the effects of autocorrelation, presence points were filtered by randomly selecting one point in each 10 km^2^ grid. Ultimately, we obtained 218 valid occurrences.

### 2.2 Climate data

To objectively test the impacts of climate change in the study area, we employed three widely applied General Circulation Models (GCMs; BCC‐CSM1‐1, CCSM4, and HadGEM2‐AO) to predict animal distributions in China (Dai et al., 2021; Ye et al., 2018). We obtained the data of 19 bioclimatic variables with a 1 km resolution from the WorldClim 1.4 database (https://www.worldclim.org/) to predict current and future species distributions. The bioclimatic variables for current species distributions were averaged over a 50‐year period (1950–2000), while those for future species distributions were averaged over 2061–2080 (referred to as 2070s hereafter). We used the most optimistic and pessimistic greenhouse gas emission concentrations for the 2070s with three Representative Concentration Pathways (RCP2.6, RCP4.5, and RCP8.5) for each GCM (Weyant, 2009).

### 2.3 Land use and land cover data

We obtained current (2020) land use and land cover change (LUCC) data from the GlobeLand30 database (http://www.globallandcover.com/) with a 30 m spatial resolution. The GlobeLand30 data were mainly developed from 30 m multispectral images and includes 10 first‐class land types, including cultivated land, forest, grass, shrub, wetland, water, tundra, artificial surface, bare land, and ice. We obtained the future (2070s) LUCC data with a 30 m spatial resolution from the database of Finer Resolution Observation and Monitoring‐Global Land Cover (http://data.ess.tsinghua.edu.cn/data/Simulation/; Li et al., 2016), which includes eight first‐class land types, including cropland, forest, grassland, shrub land, water, impervious, bare land, and snow/ice. To maintain consistency with future climate data, we selected the LUCC data under RCP 2.6, RCP 4.5, and RCP8.5 scenarios for 2070s as a representative of future land utilization types (Dai et al., 2021).

### 2.4 Additional environmental data

The Human Influence Index (HII) was obtained from the Socioeconomic Data and Applications Center, NASA (Last of the Wild, v2) at a resolution of 1 km (http://sedac.ciesin.columbia.edu/). The Digital Elevation Model (DEM) was extracted from the ASTER GDEM V2 digital elevation model with a resolution of 30 m (http://www.gscloud.cn/). As data for the HII and DEM were not available for the 2070s, we kept these two variables constant in the prediction model (Stanton et al., 2012).

### 2.5 Variables processing

To resolve the multicollinearity problem of variables, we resampled all variables to a uniform coordinate system with 1 km spatial resolution. We used the Band Collection Statistics tool to calculate the correlation coefficients between the variables. Variables with Pearson's |*r*| > .8 were excluded to eliminate multicollinearity (Appendix S1; Cord et al., 2014; Dai, 2022; Searcy & Shaffer, 2016).

### 2.6 Species distribution model

In our study, we employed the MaxEnt model to develop the habitat suitability model for the Asiatic black bear. This algorithm is widely regarded as one of the most effective approaches for predicting species distribution (Elith et al., 2011; Phillips & Dudík, 2008). With the use of only presence and environmental data, the model was able to simulate the species' current distribution in suitable habitats and predict its future distribution (Elith et al., 2011). We used MaxEnt 3.3.3k to model the current suitable habitat of the Asiatic black bear and predict the distribution of suitable habitat in response to climate and land use changes, with the following model settings: (a) 75% of the presence data of the Asiatic black bears were randomly selected as training data to build the model, while the remaining 25% were used as testing data to validate the model; (b) the Jackknife test was employed to assess the significance of environmental variables; and (c) the model performance was evaluated using the subsampling method and repeated 15 times.

Our study employed the area under the receiver operating characteristic (ROC) curve, denoted as AUC, to assess the model's performance. The AUC value ranges from 0 to 1, with a value of 1 indicating perfect model performance (Phillips et al., 2006). We utilized the Maximum Training Sensitivity Plus Specificity (MTSPS) logistic threshold value output by MaxEnt to divide the grids into suitable and unsuitable habitats for the Asiatic black bear. The grid with a value greater than the MTSPS threshold indicates that it is potentially suitable for the species (Dai et al., 2019). We converted all the Asiatic black bear distribution probability maps to binary distribution maps based on the MTSPS value. Additionally, we excluded patches with an area <10 km^2^, based on the minimum home range of the species (Nagy & Haroldson, 1990).

### 2.7 Climate refugia, altitudinal distribution and dispersal analyses

Climate refugia are defined as areas of habitat that are currently suitable for a particular species and are expected to remain so under future climate scenarios (Ashcroft, 2010; Keppel et al., 2015). These areas can provide safe havens for species to persist during times of rapid environmental change, and may offer valuable opportunities for biodiversity conservation. Identifying and protecting climate refugia has therefore become an important strategy for managing the impacts of climate change on ecosystems and species (Ashcroft, 2010; Keppel et al., 2012). In our study, we analyzed the climate refugia of the Asiatic black bear by overlaying its current distribution map with the future distribution maps in ArcGIS 10.6 (ESRI Inc.). We identified the intersection areas between the current and future distribution maps as climate refugia. The future distribution maps used in the analysis were obtained by intersecting the distribution maps generated by three GCMs and three RCPs for 2070s (Dai et al., 2021). We also analyzed the altitudinal distribution patterns of the Asiatic black bear under the current and future climate scenarios by overlaying its distribution maps with altitude layers in ArcGIS 10.6.

To identify potential dispersal paths for the Asiatic black bear between current and future suitable habitats, we used Circuitscape 4.0 (https://circuitscape.org/; McRae et al., 2013). This software simulates connectivity in heterogeneous landscapes using Circuit Theory. The landscape is represented as a conductive surface, with low resistance values assigned to landscape features that are most permeable to movement or that promote gene flow, and high resistance values assigned to movement barriers (McRae & Beier, 2007; Walpole et al., 2012). We ran Circuitscape in a paired model that iterated across all pairs in a focal node file, using suitable habitat patches as ecological nodes for each time period. We generated cumulative currents between plots for each period to visualize the state of habitat connectivity under current and future climate conditions. To simulate potential dispersal paths for the Asiatic black bear, we used the habitat suitability index (HSI) calculated by the MaxEnt model. We inverted the HSI value to link suitable distribution areas of the species with low movement resistance, and vice versa. We then used negative exponential transformation functions to convert HSI into resistance layers (Keeley et al., 2016):

IfHSI>threshold→suitable distribution areas→resistance=1

$$\begin{array}{c} \text{If}\;\mathrm{HSI}<\text{threshold}\to \text{non}\\ -\text{suitable distribution areas}/\text{matrix}\to \text{resistance}=e^{\frac{\ln\left(0.001\right)}{\text{threshold}}\times \mathrm{HSI}}\times 1000 \end{array}$$

## 3 RESULTS

### 3.1 Model performance

**FIGURE 3:**
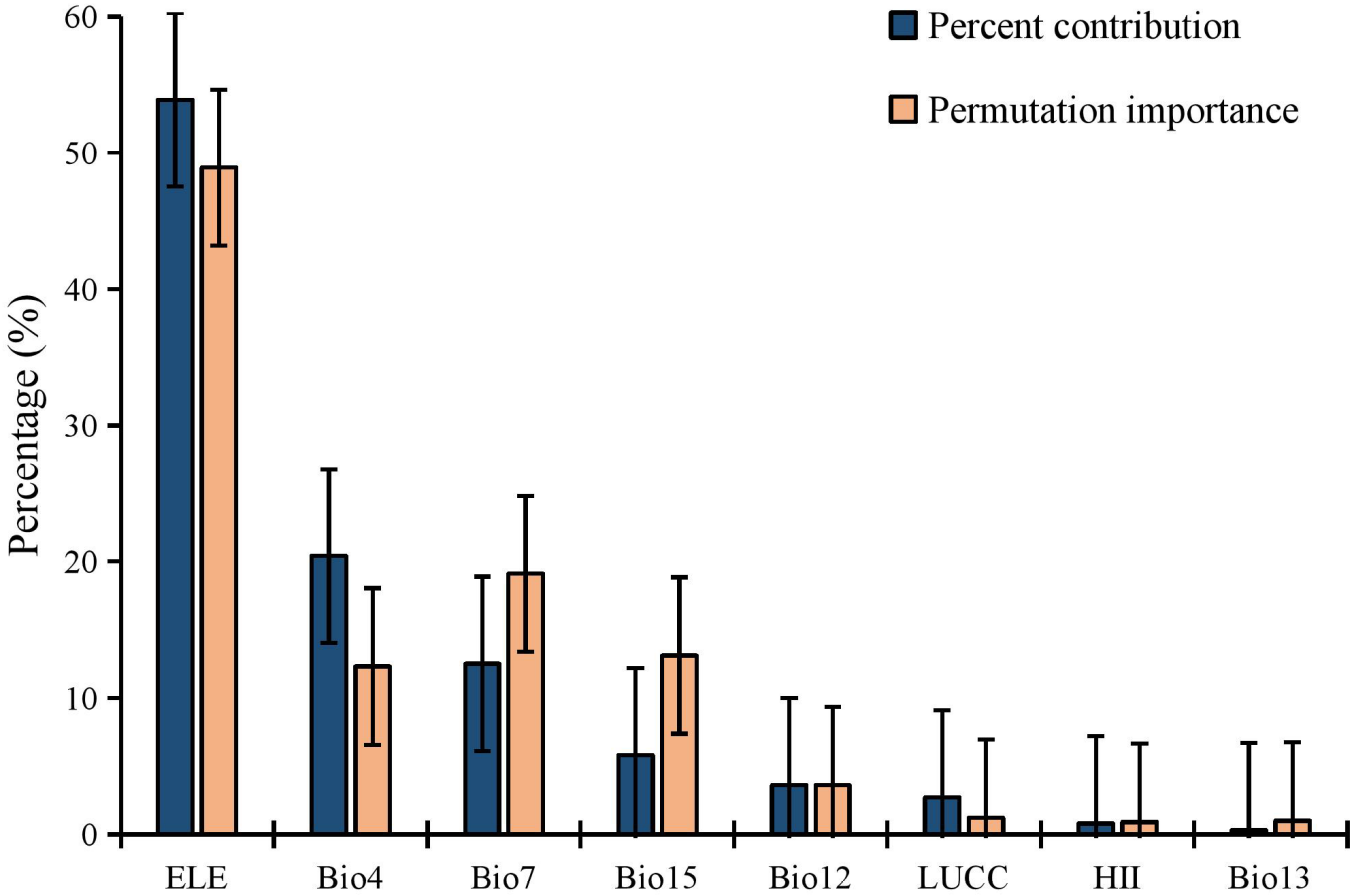
Estimates of relative contributions of the environmental variables to the MaxEnt model. Bio12, annual precipitation; Bio13, precipitation of wettest month; Bio15, precipitation seasonality; Bio4, temperature seasonality; Bio7, temperature annual range; ELE, elevation; HII, human influence index; LUCC, land use and land cover change.

After conducting a multicollinearity test, we selected eight variables with the greatest biological significance for the Asiatic black bear and used them to calculate and project the species' current and future suitable habitat distribution. The variables that remained were ELE, HII, LUCC, Bio4 (temperature seasonality), Bio7 (temperature annual range), Bio12 (annual precipitation), Bio13 (precipitation of wettest month), and Bio15 (precipitation seasonality; Appendix S2). Elevation and climatic variables were found to be the most significant, with ELE, Bio4, and Bio7 contributing 53.9%, 20.4%, and 12.5%, respectively, to the distribution of Asiatic black bear (Figure 3). To validate the model's accuracy, we used a cross‐test to evaluate the output results, and the results indicated that the model had a high accuracy, with an AUC of 0.763 (Appendix S3).

### 3.2 Projected distributions of Asiatic black bear

**FIGURE 4:**
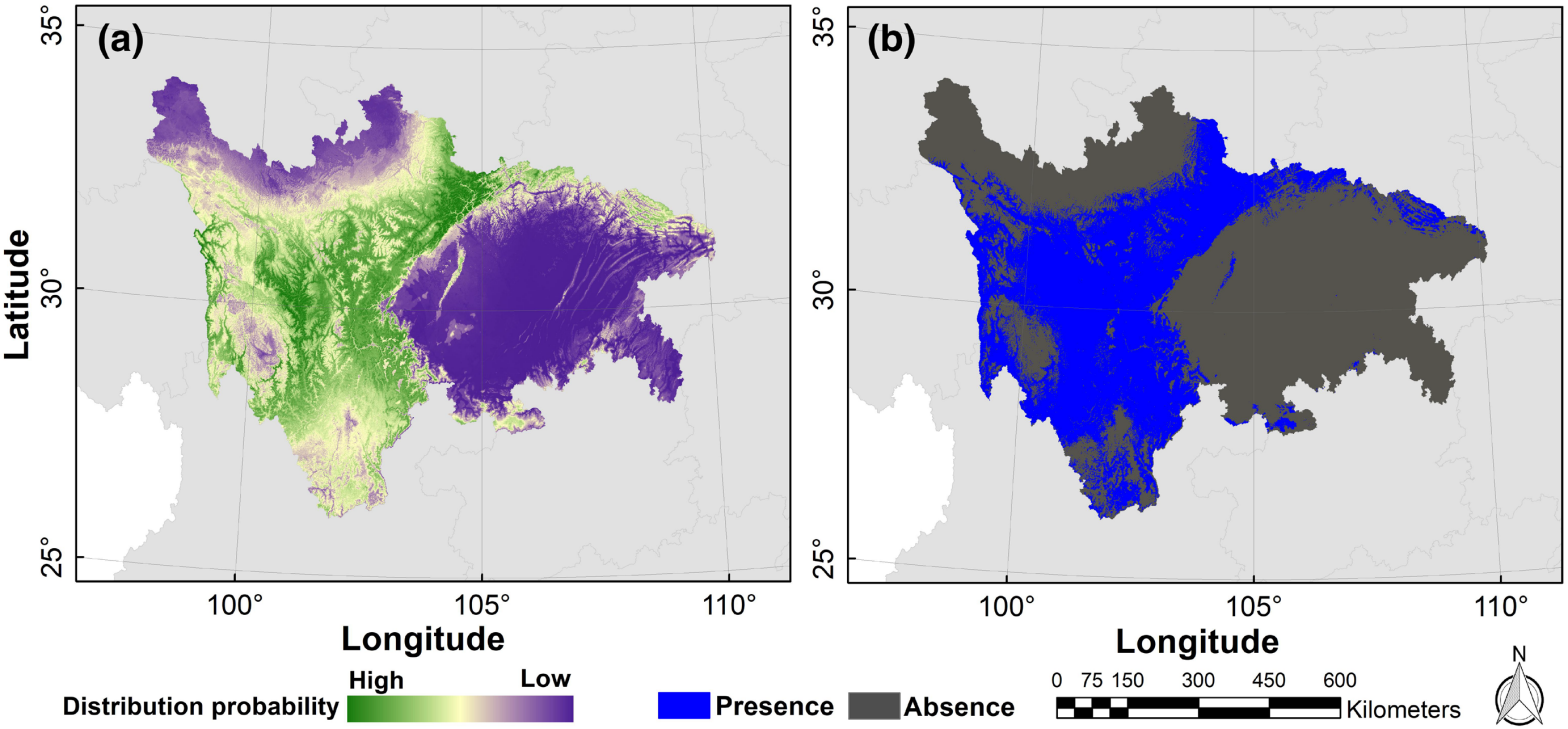
Current distribution probability (a) and binary distribution (b) of Asiatic black bear (*Ursus thibetanus*) in the study area.

**FIGURE 5:**
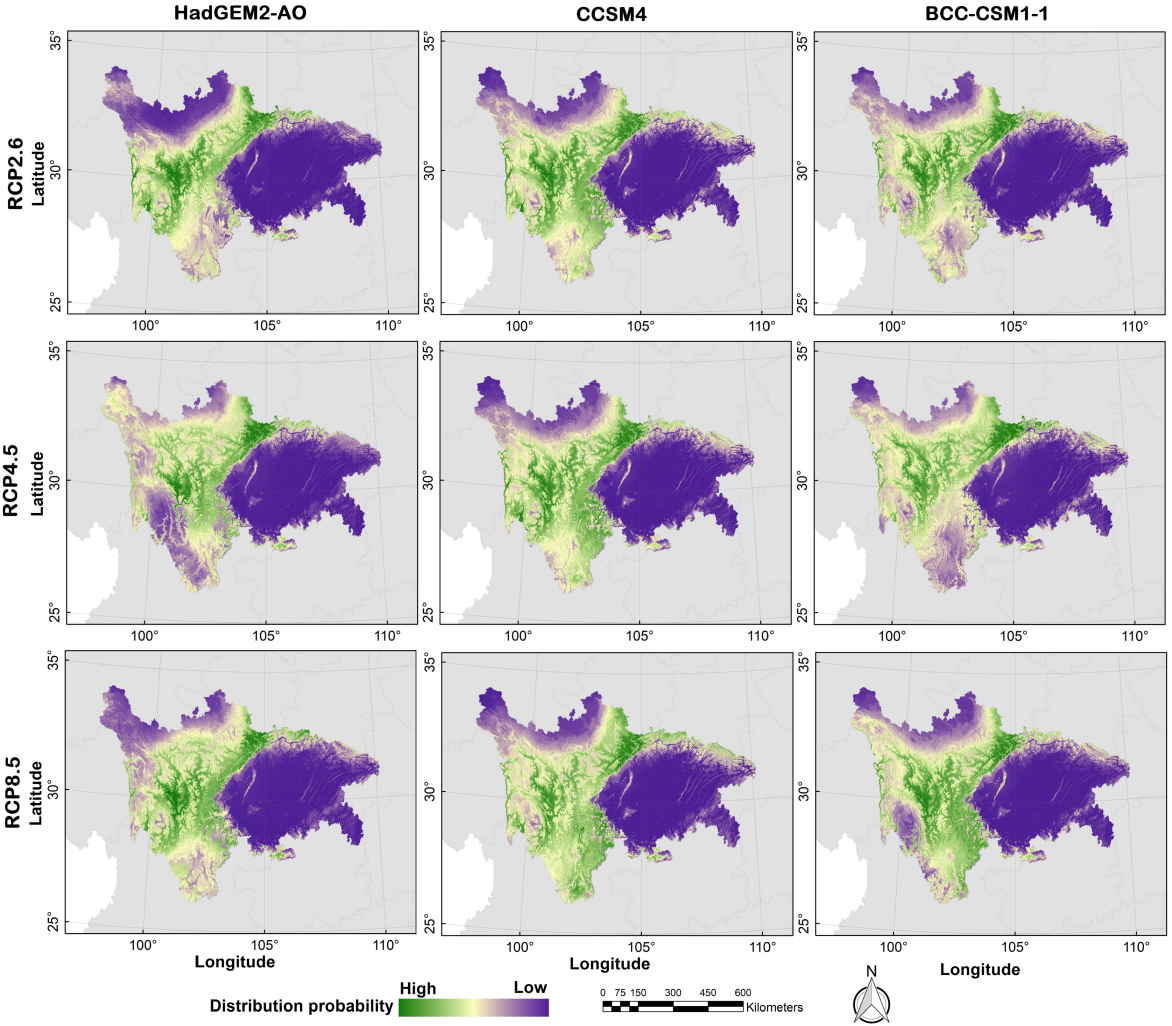
Distribution probability of Asiatic black bear (*Ursus thibetanus*) under future (scenarios of RCP 2.6, RCP 4.5, and RCP 8.5 in the 2070s) climate and land use change scenarios in the study area.

**FIGURE 6:**
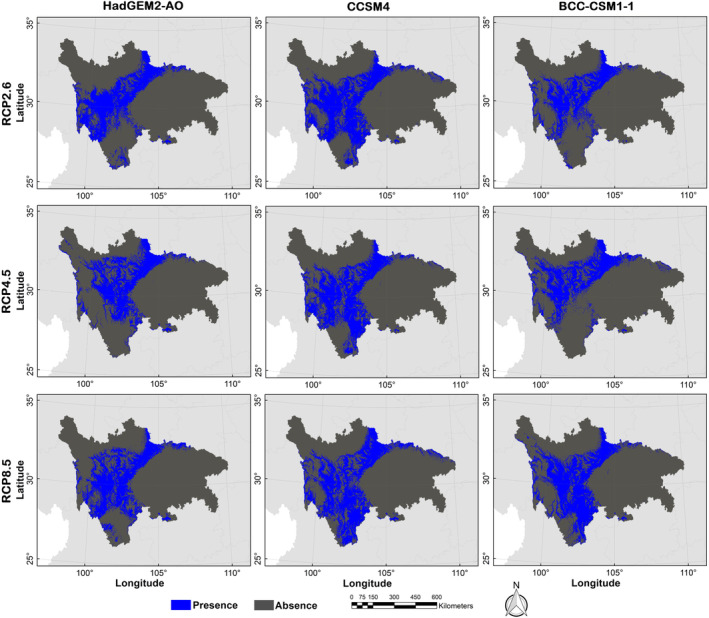
Binary distribution maps of Asiatic black bear (*Ursus thibetanus*) under future (scenarios of RCP2.6, RCP4.5, and RCP8.5 in the 2070s) climate and land use change scenarios in the study area.

**FIGURE 7:**
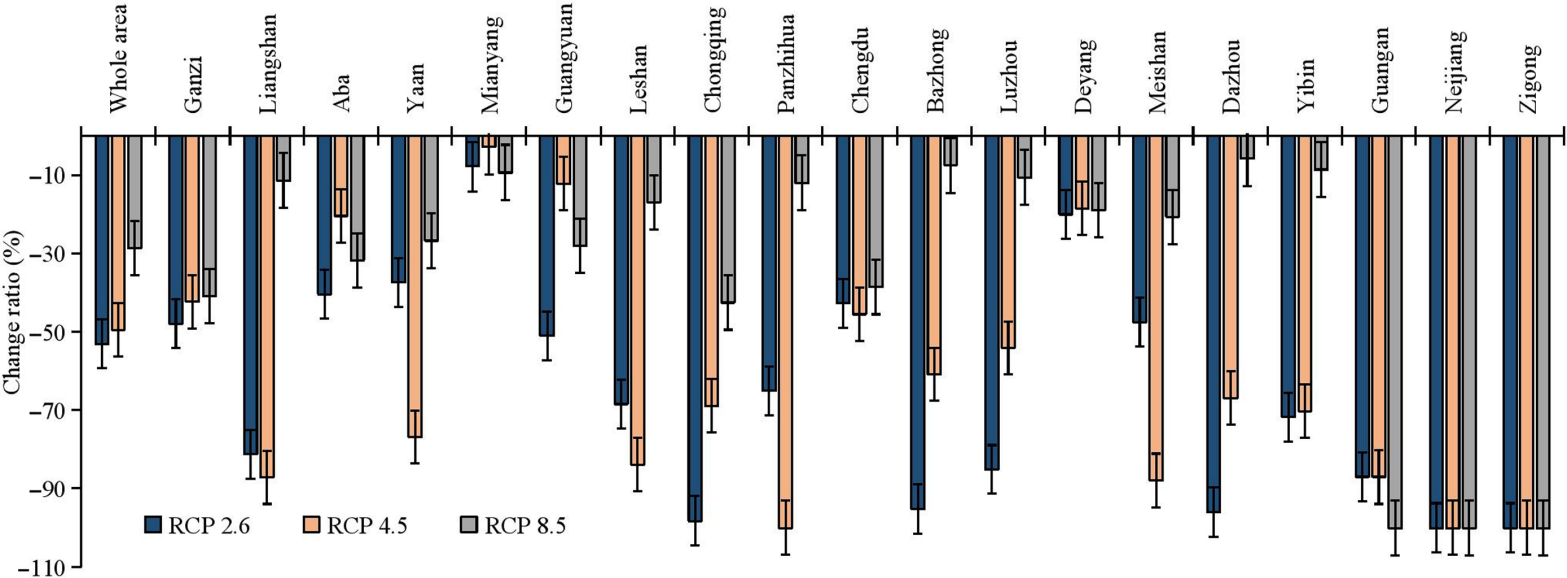
Change ratios of distributions for Asiatic black bear (*Ursus thibetanus*) under the scenarios of RCP2.6, RCP4.5, and RCP8.5 in the 2070s.

The possible distribution probabilities of the Asiatic black bear under current and future climate scenarios and land use change scenarios are shown in Figures 4a and 5. We calculated the average logistic threshold value of MTSPS for Asiatic black bear to be 0.3685 and generated binary distribution maps based on this value (Figures 4b and 6). Currently, the suitable habitat area for Asiatic black bear is 225,609.59 km^2^, mainly distributed in Ganzi, Liangshan, and Aba (Table 1). However, by the 2070s, the areas of suitable habitat were predicted to decrease. Under the RCP2.6, RCP4.5, and RCP8.5 projection scenarios, the areas of suitable habitat were estimated to be 105,821.56, 113,984.14, and 161,191.98 km^2^, respectively. The distributions of Asiatic black bear were expected to decrease by −53.1%, −49.48%, and −28.55% under RCP2.6, RCP4.5, and RCP8.5 projection scenarios, respectively (Figure 7).

**TABLE 1:** The potential distribution area of the Asiatic black bear (*Ursus thibetanus*) under current and future climate scenarios and land use changes.

Location	Current		Future					
			RCP2.6		RCP4.5		RCP8.5	
	Area (km^2^)	%	Area (km^2^)	%	Area (km^2^)	%	Area (km^2^)	%
Ganzi	86,740.09	38.45	45,198.72	42.71	50,062.12	43.92	51,355.52	31.86
Liangshan	46,510.22	20.62	8711.84	8.23	5972.77	5.24	41,236.19	25.58
Aba	39,719.33	17.61	23,680.08	22.38	31,631.2	27.75	27,126.47	16.83
Yaan	13,273.15	5.88	8312.61	7.86	3069.11	2.69	9731.11	6.04
Mianyang	10,844.69	4.81	9993.94	9.44	10,529.08	9.24	9837.80	6.10
Guangyuan	7221.70	3.20	3541.30	3.35	6345.89	5.57	5196.87	3.22
Leshan	5040.82	2.23	1589.66	1.50	808.47	0.71	4192.11	2.60
Chongqing	3503.80	1.55	63.36	0.06	1089.01	0.96	2013.96	1.25
Panzhihua	2952.24	1.31	1031.95	0.98	0.00	0.00	2600.12	1.61
Chengdu	2689.77	1.19	1542.47	1.46	1466.25	1.29	1652.58	1.03
Bazhong	1761.53	0.78	85.81	0.08	690.02	0.61	1629.06	1.01
Luzhou	1322.08	0.59	196.12	0.19	606.75	0.53	1182.27	0.73
Deyang	1310.98	0.58	1049.19	0.99	1069.64	0.94	1063.69	0.66
Meishan	1088.45	0.48	571.41	0.54	131.35	0.12	864.45	0.54
Dazhou	854.79	0.38	34.71	0.03	282.61	0.25	804.84	0.50
Yibin	771.50	0.34	217.88	0.21	229.36	0.20	704.94	0.44
Guangan	3.94	0.00	0.51	0.00	0.51	0.00	0.00	0.00
Neijiang	0.49	0.00	0.00	0.00	0.00	0.00	0.00	0.00
Zigong	0.02	0.00	0.00	0.00	0.00	0.00	0.00	0.00

*Note*: Future distributions are the intersection area of the three General Circulation Models (HadGEM2‐AO, CCSM4, and BCC‐CSM1‐1).

### 3.3 Climate refugia and altitudinal distribution of Asiatic black bear

**FIGURE 8:**
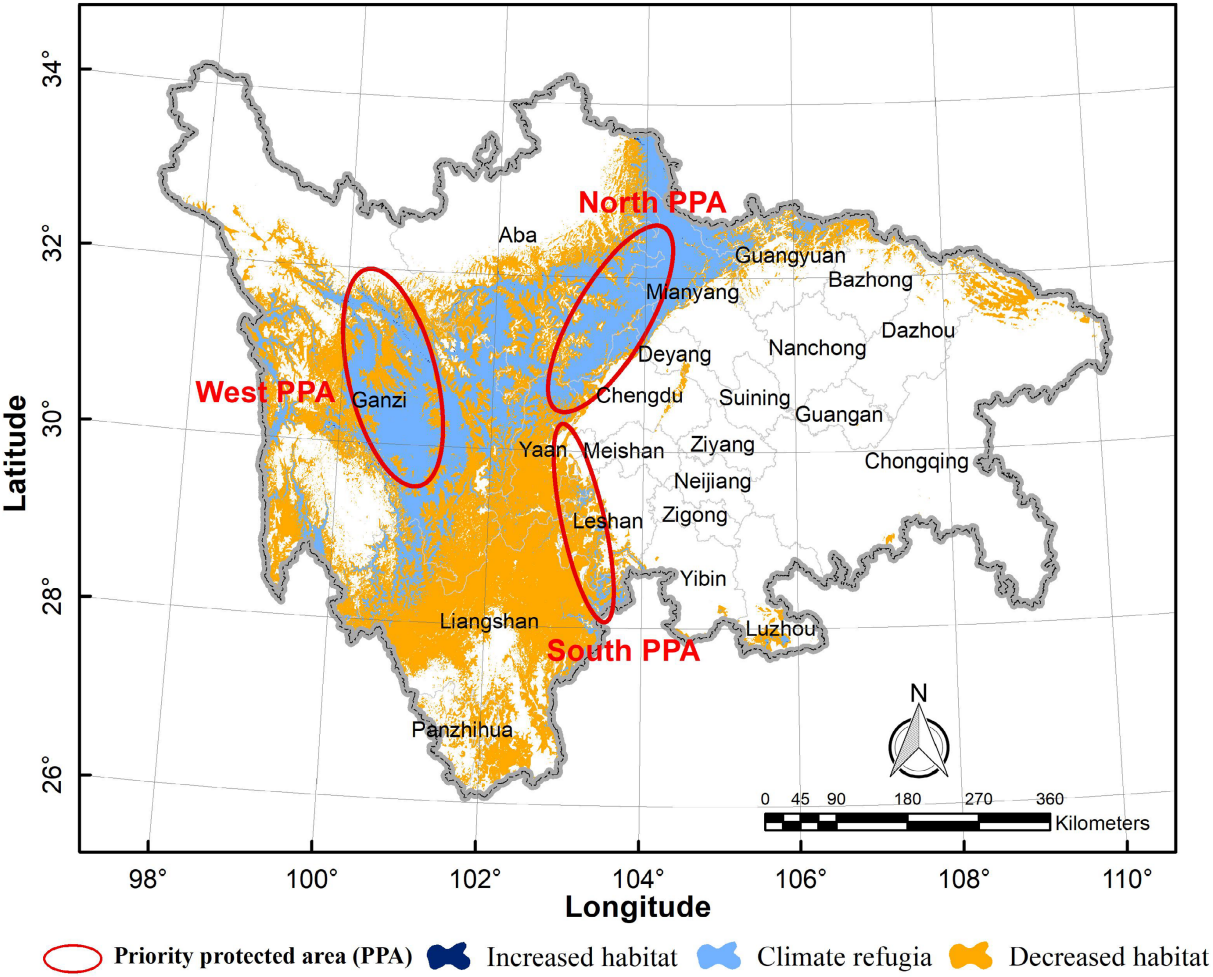
Climate refugia and priority protected area of Asiatic black bear (*Ursus thibetanus*) in the study area.

**FIGURE 9:**
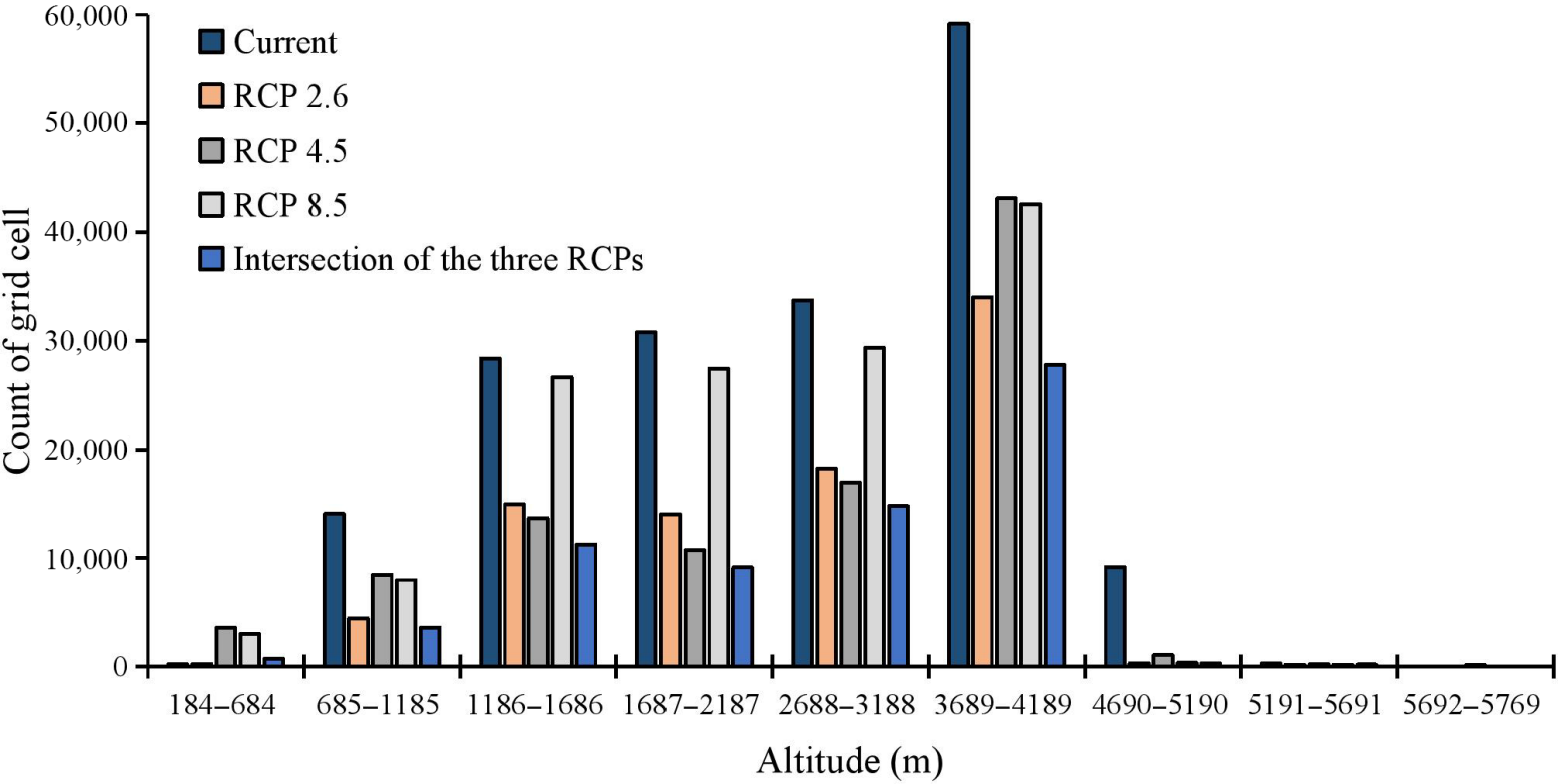
Altitudinal distribution patterns of Asiatic black bear (*Ursus thibetanus*) under current and future (the scenarios of RCP2.6, RCP4.5 and RCP8.5 in the 2070s) climate and land use change scenarios in the in the study area. Future distributions are the intersection area of the three GCMs (HadGEM2‐AO, CCSM4, and BCC‐CSM1‐1).

Areas of climate refugia and priority protected area for Asiatic black bears were mainly located in the western and northern regions of the study area, including Ganzi, Aba, Mianyang, and Liangshan, with small areas distributed in Bazhong, Dazhou, and Chongqing (Figure 8). The total area of climate refugia was about 81,226.80 km^2^, nearly half of which was primarily located in Ganzi (Table 2). Due to climate and land use changes, the distribution area of Asiatic black bears was expected to shift to higher altitudes. Currently, the highest altitudinal distribution of Asiatic black bears was 5517 m. However, under the climate scenario of RCP4.5 in the 2070s, the highest limit of altitude would rise to 5769 m, an increase of 757 m. The current and future distribution of Asiatic black bears were similar in terms of altitudinal range and were primarily distributed between 3689 and 4189 m (Figure 9).

**TABLE 2:** The climate refugia of the Asiatic black bear (*Ursus thibetanus*).

Location	Area (km^2^)	Percentage (%)
Ganzi	36,623.21	45.09
Aba	20,689.73	25.47
Mianyang	9647.29	11.88
Liangshan	4657.27	5.73
Guangyuan	3222.45	3.97
Yaan	2639.16	3.25
Chengdu	1355.60	1.67
Deyang	1020.85	1.26
Leshan	732.83	0.90
Yibin	197.89	0.24
Luzhou	189.87	0.23
Meishan	126.06	0.16
Bazhong	83.86	0.10
Dazhou	22.94	0.03
Chongqing	17.80	0.02

### 3.4 Projected dispersal paths of Asiatic black bear

**FIGURE 10:**
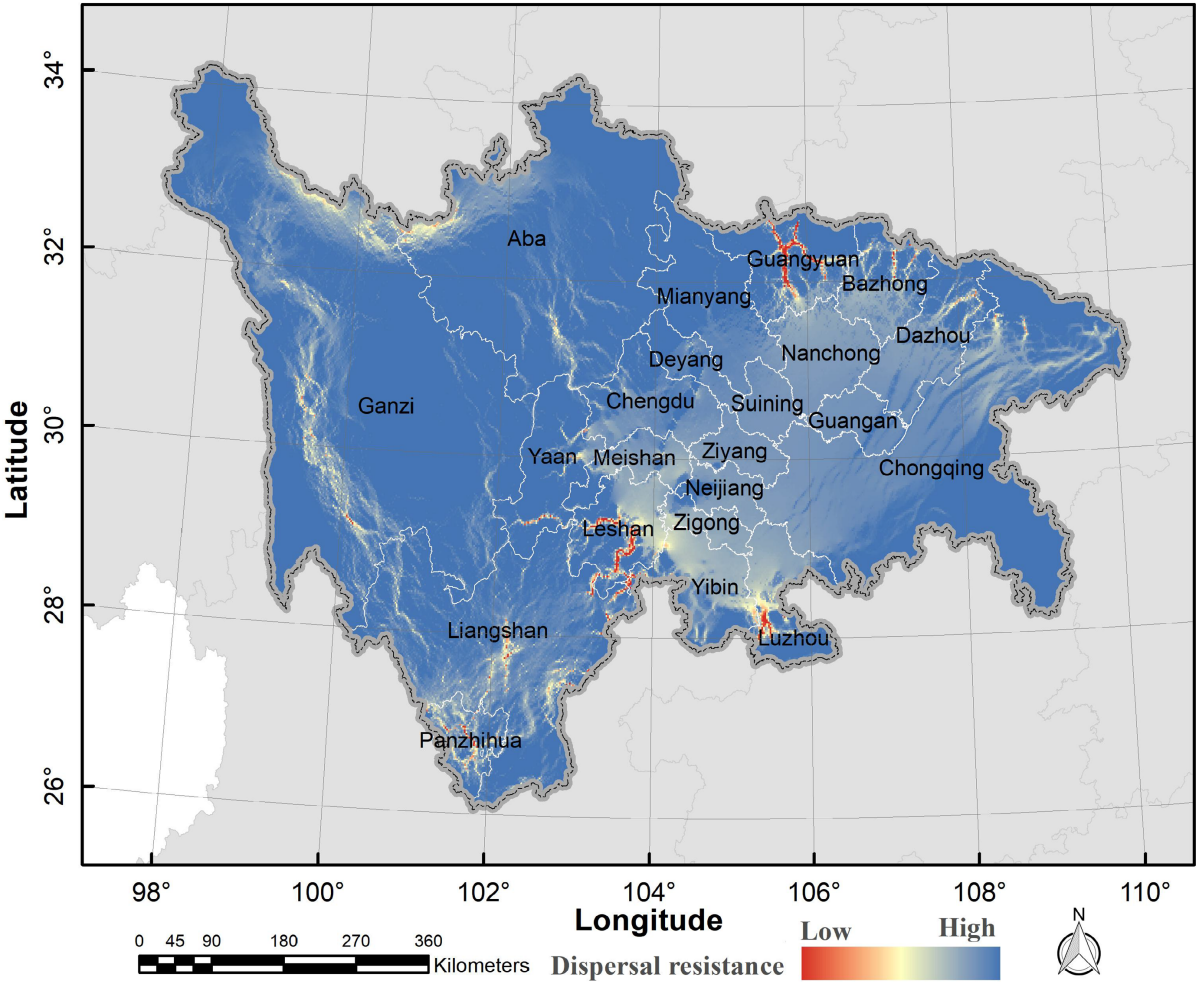
Potential dispersal paths for Asiatic black bear (*Ursus thibetanus*) under the current climate and land use change scenarios in the study area.

**FIGURE 11:**
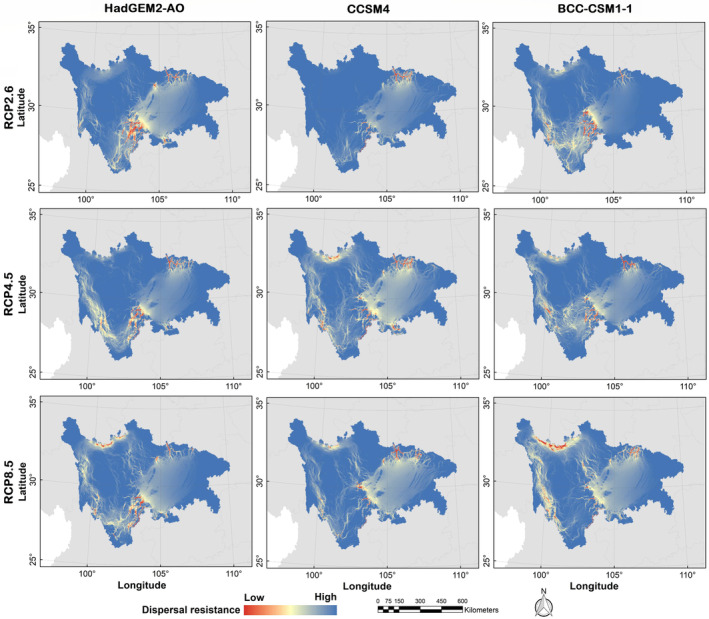
Potential dispersal paths for Asiatic black bear (*Ursus thibetanus*) under the future (the scenarios of RCP2.6, RCP4.5 and RCP8.5 in the 2070s) climate and land use change scenarios in the study area.

Based on the current climate scenario, the dispersal paths of Asiatic black bears exhibited a network pattern (Figure 10). The western, northern, and southern areas of the study site showed high density and low resistance of the dispersal paths. Notably, the western region of Ganzi displayed dense and continuous clusters of dispersal paths, making it the region with the highest habitat connectivity across the entire study area. However, the middle area featured numerous dispersal paths, albeit relatively short, with isolated and scattered ecological corridors, resulting in increased difficulty for black bears to disperse (Figure 10). In projecting toward 2070s, our findings suggested that the Asiatic black bear dispersal paths, based on various GCMs and climate scenarios, would likely experience increased resistance and decreased density (Figure 11). Specifically, the southern region of the study area was expected to demonstrate increased diffusion resistance and decreased dispersal path density. In contrast, the northern region of the study area was expected to show a gradual decline in dispersal resistance and a corresponding increase in dispersal path density, especially under the RCP8.5 scenario (Figure 11).

## 4 DISCUSSION

The ongoing development of human civilization has led to the destruction of natural habitats, resulting in the loss and reduction of Asiatic black bear populations (Bista & Aryal, 2013). Human activities have significantly and negatively impacted the habitat of the Asian black bear, resulting in disturbances and the fragmentation of its natural habitat (Ahmad et al., 2022; Escobar et al., 2015). Moreover, poaching poses a severe threat to the Asian black bear population, and has caused a decline in its numbers (Ullah et al., 2020). Currently, these wild animals are under strict legal protection, many of the factors that once threatened the survival of the Asian black bear have been eliminated. However, climate change and human activities are presently identified as the primary factors that contribute to the degradation and fragmentation of the Asian black bear's habitat (Escobar et al., 2015; Zahoor, Liu, Ahmad, et al., 2021). In particular, the impacts of future climate change present a challenge to the conservation of Asiatic black bears (Zahoor, Liu, Ahmad, et al., 2021). To address these challenges, habitat vulnerability assessment with regard to climate change and land use change is essential for mitigating the negative impacts of climate change on the species (Usman et al., 2023). Predicting habitat succession and dispersal paths, and devising proactive protection measures can increase the preservation of living space for Asiatic black bears and bolster their ability to adapt to future climate change. Such efforts may provide a sound foundation for human response to global climate change and conservation of the Asiatic black bear (Doko et al., 2011; Usman et al., 2023).

Even under a moderate greenhouse gas emissions scenario (PCR4.5), climate change is projected to reduce the current suitable habitat area of the Asiatic black bear by 49.48%, with the most significant impact occurring in the southern region of the species' distribution range, such as Panzhihua and Liangshan, where habitat connectivity is low. These changes will accelerate the fragmentation of Asiatic black bear habitat in the region, posing a serious threat to the species' survival and reproduction. Therefore, proactive conservation measures, such as habitat restoration and the construction of ecological corridors, are necessary to mitigate the adverse effects of climate change and support the dispersal of Asiatic black bear populations into the western and northern mountains. Our model predicted that climate change will expand the distribution of suitable habitats for Asiatic black bears in eastern Ganzi and central Aba. However, human activities, including construction and grazing, hinder the dispersal of Asiatic black bears into these areas to some extent, thereby limiting their potential to take advantage of the newly available habitats. Therefore, ecosystem management projects aimed at promoting habitat restoration are essential to improve habitat area and connectivity and facilitate the migration of more Asiatic black bears into these future habitat areas.

Based on the characteristics of the Asiatic black bear species, we have identified climate refugia and ecological corridors that are essential for its population maintenance. The primary climate refugia for this species are located in Ganzi, Aba, and Mianyang. However, due to the impact of climate and land use change, Asiatic black bears will be forced to migrate from currently unsuitable habitat to more suitable ones in the future. To facilitate this migration, we can utilize the dispersal path predicted by the Circuit Theory model as an ecological corridor for the transfer of the Asiatic black bear (Figure 10). The cluster area for dispersal paths in western Ganzi is crucial for the current dispersal of the Asiatic black bear. Additionally, the cluster area for dispersal paths in northern Liangshan and central Aba is of greater significance to enable the species to track future climate changes. Therefore, recent conservation efforts can focus on western Ganzi by assessing habitat quality and restoring degraded habitats. Priority protection should be given to the future dispersal path areas located in northern Liangshan and central Aba to maintain ecosystem integrity in this region and meet the future dispersal needs of the Asiatic black bear. In addition, habitat quality monitoring should be strengthened in these areas, including bear population dynamics, plant community dynamics, phenology, and food availability.

Assessing the impacts of climate change on wildlife is essential to develop effective adaptation strategies for conservation (Galatowitsch et al., 2009; Midgley et al., 2002; Nunez et al., 2019). However, many protected areas were established without adequate consideration of the potential impacts of climate change on wildlife, and as a result, the conservation effectiveness of these areas may be reduced or lost (Hannah et al., 2002; Mackey et al., 2008). Our study revealed that the existing protected area system is insufficient to protect the suitable habitat of Asiatic black bears, as most of their suitable habitat lies outside the protected areas, and many of these protected areas will be affected by climate change, thus reducing their protective capacity. To address this issue, we propose the need to enhance the adaptation capacity of the protected areas in Ganzi, Mianyang, Aba, and Liangshan to cope with climate change, as these areas possess a significant number of Asiatic black bear climate refugia. Nevertheless, most of these climate refugia are isolated from the protected area network within the study area. Our study predicted that the suitable habitat for Asiatic black bears would expand in and around these areas, emphasizing the need for these areas' effective management and monitoring to ensure their suitability as a future refuge for this species.

Assessing the vulnerability of habitat is helpful for conservation biologists to understand which regions will experience changes under future climate change scenarios, and to formulate biodiversity conservation recommendations for adapting to such changes (Pacifici et al., 2015; Watson et al., 2013). The results of the habitat vulnerability assessment indicated that priority conservation measures should focus on areas that are losing habitat, as these areas will be greatly impacted by climate change, leading to a significant loss of suitable habitats and posing a serious threat to the survival of Asian black bears in the future. Additionally, attention should be paid to areas where suitable habitats have not changed or are newly added in the future, as these areas will become potential refugia in the face of climate change. The Asian black bear is a umbrella species for global biodiversity conservation, and its protection is a critical contribution and responsibility of the Chinese government to the global conservation effort. Vulnerability assessments of endangered species in the context of climate change have been identified as a priority action plan for the next 20 years in China's Biodiversity Conservation Strategy and Action Plan (2011–2030), and the Chinese government plans to integrate and optimize protected areas in Sichuan province, Chongqing municipality, Gansu province, and other areas to further protect the Asian black bear and sympatric species.

Our study conducted a habitat and dispersal vulnerability assessment of Asiatic black bears in the context of climate and land use changes, and proposed scientific strategies to enable them to adapt to climate change, providing a reference for the conservation of Asiatic black bears and sympatric species. Based on the findings, we proposed some specific recommendations for the future conservation of Asiatic black bears in response to climate change: (a) Establish new protected areas. Protected areas serve as the foundation for wildlife conservation and are crucial for species protection. In the study area, the current protection of suitable Asiatic black bear habitats within the existing protected area system is insufficient, necessitating further improvement. Establishing new protected areas will help enhance the conservation of Asiatic black bear populations and their habitats. (b) Adjust protected area boundaries. As climate change expands the distribution of suitable Asiatic black bear habitats in certain protected areas, it becomes necessary to adapt the boundaries of these areas. The scope of these protected areas should be adjusted to encompass suitable habitats in the surrounding regions, with particular focus on areas like Aba and Ganzi. This adjustment will ensure that the protected areas effectively cover the habitats necessary for black bears under changing climatic conditions. (c) Protect climate refugia. Given the significant projected decrease in suitable habitat for the Asiatic black bear across all three projection scenarios, safeguarding climate refugia becomes a critical priority. Climate refugia are areas expected to maintain suitable conditions for black bears despite changing climatic conditions. Identifying and protecting these refugia will be vital to ensuring the long‐term survival of black bear populations in the face of habitat loss. (d) Preserve ecological corridors. The anticipated shift of distribution areas and dispersal corridors to higher altitudes implies that black bears will need to relocate to find suitable habitats. To facilitate their movement and gene flow between populations, the preservation of ecological corridors becomes essential. By protecting and maintaining these corridors, habitat fragmentation can be minimized, enabling the connectivity and genetic diversity necessary for the survival of black bear populations.

## 5 CONCLUSION

In the context of global climate change, identifying priority areas for biodiversity conservation has become a core component of national strategies for protecting biodiversity (Summers et al., 2012). Vulnerability assessment is an important aspect of studying the effects of climate change on wild animals, and identifying climate refugia and dispersal paths is key to adapting to and mitigating the impacts of climate change (Dai et al., 2021; Foden et al., 2019). The purpose of conducting habitat assessments of wild animals under the backdrop of climate change is to determine vulnerable species and the factors contributing to their vulnerability (Preston et al., 2011; Thuiller et al., 2006). The results of these assessments help humans understand the effects of climate change on wild animals and provide a scientific basis for the formulation of protection measures to help them adapt to climate change (Foden et al., 2019; Summers et al., 2012; Watson et al., 2013). When prioritizing plans for biodiversity protection, it is important to consider studying how climate change affects endangered species as a top priority. Therefore, in this study, we selected a representative endangered species, the Asiatic black bear, from the Sichuan‐Chongqing Region of China. We used ecological niche models to predict the impact of climate change on its suitable habitat distribution and to determine the current and future distribution patterns of this endangered species. We assessed the risks faced by endangered species in the context of climate change and identified climate refugia. Furthermore, we used the Circuit Theory model to construct Asiatic black bear movement corridors based on changes in suitable habitats to increase their ability to adapt to climate change. We hope that the consideration of climate change will become an integral part of future strategies for biodiversity conservation, allowing us to more effectively and sustainably protect biodiversity by developing dynamic conservation strategies.

## AUTHOR CONTRIBUTIONS

**Yunchuan Dai:** Data curation (equal); formal analysis (equal); funding acquisition (equal); investigation (equal); methodology (lead); resources (equal); software (equal); supervision (equal); validation (equal); visualization (equal); writing – original draft (lead); writing – review and editing (lead). **Heqing Huang:** Data curation (equal); resources (equal); writing – review and editing (supporting). **Yu Qing:** Writing – review and editing (supporting). **Jiatong Li:** Writing – review and editing (supporting). **Dayong Li:** Conceptualization (lead); funding acquisition (lead); investigation (lead); supervision (lead).

## FUNDING INFORMATION

This research was supported by the Second Tibetan Plateau Scientific Expedition and Research Program (Grant/Award Number: 2019QZKK0501), the Youth Innovation Research Team Project of Chongqing Academy of Social Sciences (Grant/Award Number: 2022D0307), and the Higher Education Scientific Research Project of the Education Department in Guizhou Province, China (Grant/Award Number: Qianjiaoji [2022] 365).

## Supporting information


Appendix S1.
Appendix S2.
Appendix S3.

## Data Availability

We used open‐access data from WorldClim (http://www.world clim.org/; https://doi.org/10.1002/ece3.3994), ASTER GDEM V2 (http://www.gscloud.cn/; https://doi.org/10.7717/peerj.3477), and Last of the Wild, v2 (http://sedac.ciesin.columbia.edu/; https://doi.org/10.7717/peerj.3477).
